# Midwives’ perceptions on using a fetoscope and Doppler for fetal heart rate assessments during labor: a qualitative study in rural Tanzania

**DOI:** 10.1186/s12884-018-1736-y

**Published:** 2018-04-16

**Authors:** Paschal Francis Mdoe, Hege Langli Ersdal, Estomih Mduma, Robert Moshiro, Hussein Kidanto, Columba Mbekenga

**Affiliations:** 1 Haydom Lutheran Hospital, Mbulu, Tanzania; 2 University of Stavanger, Stavanger, Norway; 3 Department of Anesthesiology and Intensive Care, Stavanger University Hospital, Stavanger, Norway; 4 Muhimbili National Hospital, Dar es Salaam, Tanzania; 5 Aga Khan University, School of Nursing and Midwifery, Kibaha, Tanzania; 6 Department of Research, Stavanger University Hospital, Stavanger, Norway; 7 Haydom, Tanzania

**Keywords:** Midwives, Fetoscope, Doppler, Fetal heart rate, Labor

## Abstract

**Background:**

The Doppler is thought to be more comfortable and effective compared to the fetoscope for assessing the fetal heart rate (FHR) during labor. However, in a rural Tanzanian hospital, midwives who had easy access to both devices mostly used fetoscope. This study explored midwives’ perception of factors influencing their preference for using either a Pinard fetoscope or a FreePlay wind-up Doppler for intermittent FHR monitoring.

**Methods:**

Midwives who had worked for at least 6 months in the labor ward were recruited. Focus group discussion (FGD) was used to collect data. Five FGDs were conducted between December 2015 and February 2016. Qualitative content analysis was employed using NVivo 11.0.

**Results:**

Three main themes emerged as factors perceived by midwives as influencing their preference; 1) Sufficient training and experience with using a device; Midwives had been using fetoscopes since their midwifery training, and they had vast experience using it. The Doppler was recently introduced in the maternity ward, and midwives had insufficient training in how to use it. 2) Ability of the device to produce reliable measurements; Using a fetoscope, one must listen for the heartbeat, count using a watch, and calculate, the Doppler provides both a display and sound of the FHR. Fetoscope measurements are prone to human errors, and Doppler measurements are prone to instrumental errors. 3) Convenience of use and comfort of a device; Fetoscopes do not need charging, and while it is possible to “personalize/hide” the measurements, and may be painful for mothers. Dopplers need charging and do not cause pain, but provide limited privacy.

**Conclusion:**

Midwives’ preferences of FHR monitoring devices are influenced by the level of device training, experience with using a device, reliable measurements, and convenience and comfort during use. Fetoscopes and Dopplers should be equally available during midwifery training and in clinical practice.

**Electronic supplementary material:**

The online version of this article (10.1186/s12884-018-1736-y) contains supplementary material, which is available to authorized users.

## Background

Incidences of fresh stillbirths and intrapartum-related asphyxia are still unacceptably high in low-resource settings [1]. Globally, each year, an estimated 1.3 million babies are fresh stillbirths, and 700,000 early neonatal deaths occur due to birth asphyxia [2–6]. These perinatal deaths may be due to hypoxic-ischemic encephalopathy because of interrupted placental blood flow. To prevent intrapartum asphyxia, early detection of fetal responses to fetal hypoxemia, as indicated by fetal heart rate (FHR) abnormalities, is crucial [7, 8]. Ersdal et al. described the relationship between intermittent auscultation of FHR using a standard fetoscope and perinatal outcome. Detection of an absent or abnormal FHR was associated with fresh stillbirth or birth asphyxia, with increased need of neonatal resuscitation. In addition, as many as 40% of the babies who ended up as a fresh stillbirth had a normal or abnormal FHR on admission. These findings may reflect an inability to perform measurements correctly or as often as recommended using the fetoscope [8]. However, intermittent auscultation of FHR, if performed as often as recommended, is regarded as safe and effective in low-risk pregnancies and birth [9–11].

In high-income countries, continuous FHR monitoring is readily available, using high- technology devices such as a cardiotocograph (CTG). These devices are costly and not available or feasible in low-resource settings. A recently Cochrane review found that continuous use of CTG did not improve perinatal survival rather contributed to the increased cesarean section births [12].The intermittent FHR monitoring using hand-held Doppler ultrasound found to detected more intrapartum FHR abnormalities as compared to the Pinard fetoscope [11, 13]. Despite the findings in these studies, FHR assessments using fetoscopes have been the most common method of fetal monitoring in low-resource settings, because Dopplers are costly, and not usable in settings without reliable electricity or available batteries. Nevertheless, the Pinard fetoscope is reported to be difficult to use, time-consuming, and painful for the mother, whereas the Doppler is believed to be less painful, easier to handle, and more reliable [14]. Mangesi et al. assessed laboring women’s preferences, and found that a standard hand-held Doppler monitor was most acceptable, followed by the Pinard fetoscope, and finally the CTG [15].

Studies from Tanzania have also revealed that intermittent FHR auscultations using fetoscopes are not conducted according to guidelines, and that this may cause unnecessary perinatal deaths and illness [16, 17]. Based on these findings, a new affordable Freeplay Doppler was developed to meet the needs of rural settings [18]. This Freeplay Doppler was randomly tested against the Pinard fetoscope in Uganda and in our hospital in rural Tanzania [13, 19]. The study from Uganda revealed an increased detection rate of FHR abnormalities in the Freeplay Doppler arm, but this did not translate into improved perinatal outcomes [13]. Our study under the Safer Births project, in a rural hospital, found that abnormal FHR detection was similar between the Pinard fetoscope and Freeplay Doppler, but midwives often broke the randomization protocol by using the Pinard fetoscope instead of the Freeplay Doppler. The Safer Births project which focuses on the improvement of FHR monitoring and newborn resuscitation has been running in the hospital since 2009. The research assistants in this project have observed all births since 2009, and recorded the most commonly used device for each woman regardless of the randomization arm, and the majority of the midwives seemed to prefer the Pinard fetoscope [19]. This contradicts with findings from other studies where professionals preferred the Doppler over the fetoscope [13, 15].

For FHR monitoring to be effective, the assessments must be performed correctly, the results must be interpreted satisfactorily, and the interpretation must provoke appropriate and timely responses [11]. Correct FHR monitoring, proper interpretation, and acting on the information depend on the midwives’ perception of the device used for monitoring. Smith et al. documented that professionals preferred a device which gives reliable results, so that providers are certain they are able to adequately communicate the findings and make decisions [20].

**Fig. 1:**
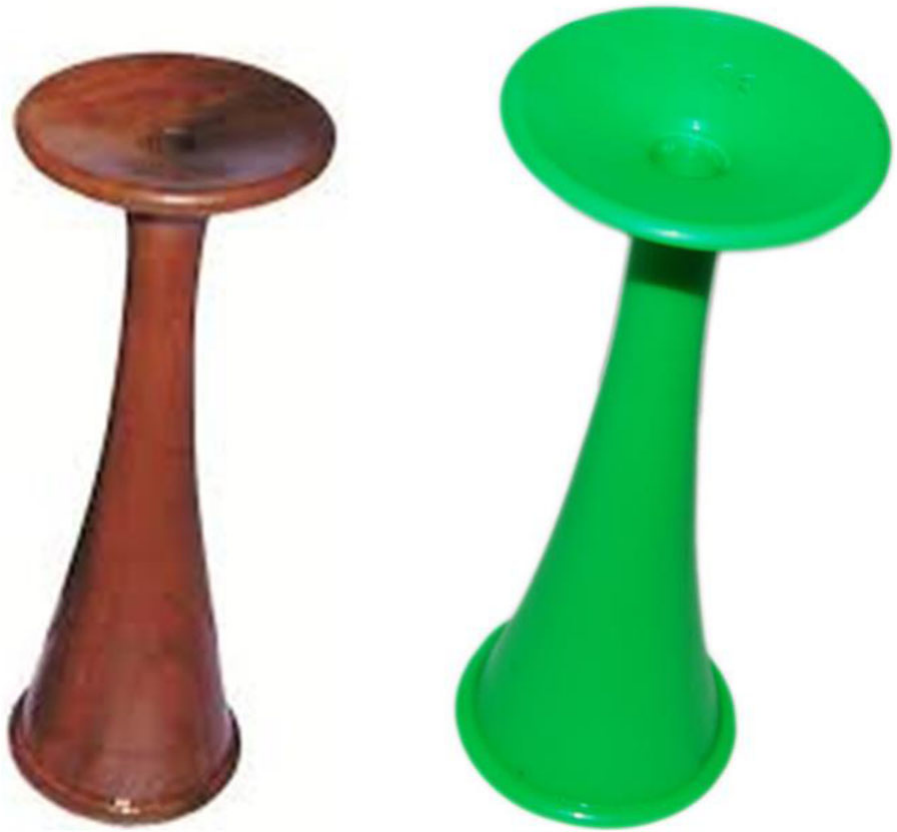
Pinard fetoscopes

**Fig. 2:**
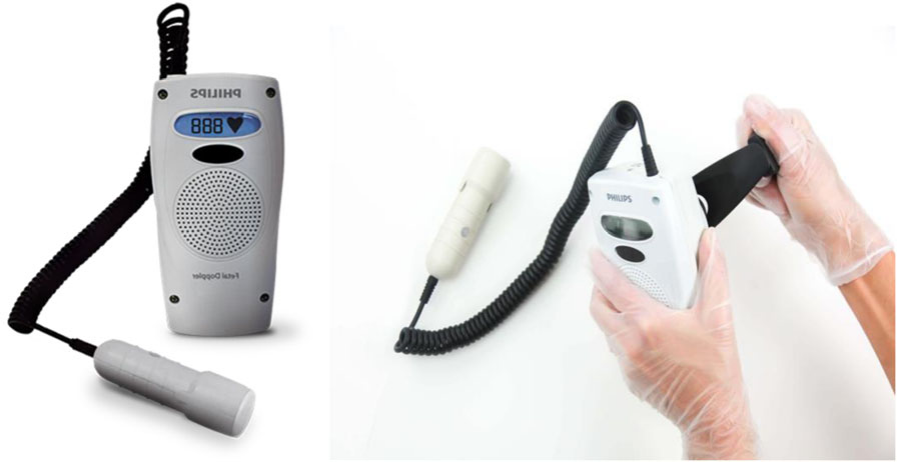
Hand held Doppler

Fetoscope is a hollow horn, made up of wood, plastic or metal, normally about 8 in. or more. It amplifies sound similar to an ear trumpet from the fetal heart, via a bone construction to the midwife’s ear (Fig. 1). Hand-held Doppler is ultrasound with a transducer used to detect the fetal heart beats. It produces audible simulation of the heart beats and displays the heart rate in beats per minute. It needs battery/charging to function (Fig. 2).

Studies have evaluated the effectiveness of devices for FHR monitoring [11, 13]. However, very little research has been done to elucidate users’ perspectives and to better understand the users’ opinions, perceptions, and attitudes towards different devices and methods. These human factors probably have a considerable impact on how FHR monitoring is performed, and thus on the interventions taken and the baby’s outcome. Typical hindrances and factors perceived by health professionals when monitoring FHR using both Dopplers and fetoscopes are still poorly defined.

The aim of this study was to explore midwives’ perceptions on factors influencing their choice of using either the Pinard fetoscope or Freeplay Doppler for intermittent FHR assessments in a rural low-resource setting.

## Methods

### Study setting

The study was conducted at Haydom Lutheran Hospital located in rural Northern Tanzania, 300 km from the nearest urban center, with a poor rural population in the catchment area. The hospital has a well-established infrastructure for collaborative research and data collection. It is the referral hospital for approximately 500,000 people, while the greater reference area covers about 2 million people. The hospital has about 35 midwives working in the maternity ward, with about half of them mainly allocated to the labor ward. The hospital has about 45% staff turnover yearly, which may be due to its rural location. Few midwives have more than 10 years’ experience working in the labor ward. A senior midwife is in-charge of the laboring suite and oversees the daily functioning and conduct of midwives. A senior obstetrician oversees the daily functioning of all doctors in the ward and do teaching sessions for both junior doctors and midwives. Midwives are responsible for managing normal pregnancy, labour and delivery, while doctors in consultation with obstetricians are responsible for supporting midwives to conduct difficult births and perform cesarean sections. The hospital has an average of 4500 births per year, which is about 53% of women giving birth in the catchment area, less than 10% give births at other health facilities, and the rest give birth at home without help of a skilled attendant. Both women with complicated and uncomplicated labors give birth at the hospital with an approximate 21% cesarean section rate. FHR monitoring during labor was mainly performed intermittently using a fetoscope, and the Freeplay Doppler, which was introduced in 2013 as part of a randomized controlled study, there was no facilities for continuous FHR monitoring in the hospital. All midwives were trained in the general operation of Freeplay Doppler. The training given was a half day training done twice during the randomization study time as a refresher for all, but all new midwives had a formal training during the orientation time. The training focused on how to use the device in general, charging, interpreting the displays and how to position the transducer in order to get the proper FHR and they were reminded about general labor management.

### Study design

An inductive qualitative design employing focus group discussion (FGD) was conducted between December 2015 and February 2016. FGD were used in order to explore midwives’ own perception and opinions of FHR monitoring using different devices. FGD refers to a method of data collection that gathers people of similar backgrounds to discuss a research topic [21–23].

### Sample size and sampling

In order to recruit informants with different work experiences, a purposive sampling technique (add reference) was used. Midwives with at least six months’ duration or more of working in the labor ward, were recruited to participate in the FDG. Recruitment was performed by the first author (PM), who is a MD (obstetrician) and PhD student, in collaboration with a senior midwife of the maternity ward. Five midwives with varying work experience (years of midwifery practice) were recruited for each group, therefore a total of 25out of 35 midwives met the inclusion criteria and included in the FDG. The purpose of mixing different work experiences was to allow for a variety of perceptions and more lively discussion [24].

We allowed only five participants in each FGD to maximize the involvement of each participant and enhance participation during the discussion.

### Data collection and handling

We conducted one pilot interview to see how well the interview guide was understood and captured the essence of conducting this study. The pilot FGD, which was moderated by PM and an assistant moderator (who is a clinical psychologist experienced in conducting interviews and FGDs), led to minor modification of the interview guide to better capture adequate information and flow of the discussion. The FGDs were audio-recorded using a digital recorder. The assistant moderator took notes and recorded the discussion while the moderator led the discussion.

At the end of each FGD, PM together with the moderator reviewed the information collected to advance their understanding regarding the midwives’ use of the two devices for FHR monitoring. Data collection stopped after five FGDs, when the saturation point was reached [24]. The audio recordings were transcribed and translated from Kiswahili to English for analysis.

### Data analysis

A pragmatic method of qualitative content analysis was used to understand the attributions of midwives concerning the use of the two devices for FHR monitoring [22, 25]. The first and fourth authors first read all the transcripts (open coding) and coded the first transcript manually; then they shared codes with the other authors. Then, the preformed codes were entered into the computer software (NVivo 11.0), which was used to code the rest of the transcripts. This software was used to sort and organize codes into categories [26]; similar categories were manually merged to form themes, and three main themes were identified. Table 1, serves as an example.

**Table 1:** Shows the example of FDG analysis

FGD quote	Initial coding	Final code	Theme
(FGD5. R4). When I am in hurry and I use Pinard to monitor fetal heart rate, there is a risk of not counting beats correctly [Mmh] Sometime when I am in hurry, I fail to count them correctly and identify if they are abnormal. It is difficult to discover it quickly with Pinard. But with Doppler, it displays the measurements quickly. It is easy to see the heart rate if they are low or high and I can repeat auscultation. But with Pinard, I can’t easily repeat. [Mmh] Pinard is difficult to use in a busy ward	∙ Human error in counting ∙ Possibility of missing abnormality ∙ Can’t display measurement	Pinard measurements are subjective	Reliability of measurements
	∙ Doppler displays measurements ∙ Possibility of reproducing measurements	Doppler measurements are objective	

### Ethical considerations

This study is part of the Safer Births project, which has been approved by Haydom Lutheran Hospital, and certified by the National Institute for Medical Research (NIMR), Tanzania and the Regional Committee for Medical and Health Research Ethics, Western Norway (REK Vest).

Written informed consent was obtained from all participants. Each participant in each FGD was given an identification number between 1 and 5 (labeled R1, R2…. R5), in order to hide the identity of participants in the audio recordings. The FGDs were conducted within the hospital premises in a private room at a convenient time for those midwives who were off duty. Before the FGD, all participants were informed that the data collected were to be kept confidential and access was restricted to the researchers only. Data recorded during the FGDs were stored in two different hard disk servers to ensure security.

## Results

The mean age of the participants was 32.4 years, and the mean time of working in the labor ward was 3.48 years (Table 2).

**Table 2:** Participants’ age and years of working in the labor ward

Age of Participants (years)	Frequency
20–29	11
30–40	10
> 40	4
Years working in labor ward (years)	Frequency
1–5	19
> 5	6

The overall aim of this study was to explore midwives’ perceptions of factors influencing their choice of using either a Pinard fetoscope or a Freeplay Doppler for intermittent FHR monitoring. Three main themes emerged from the FGDs (Table 3), and these are presented with illustrative quotes from the FGD discussions.

**Table 3:** Themes of midwives’ perceptions of factors influencing their choices of using either a Pinard fetoscope or a Freeplay Doppler for FHR monitoring

1	Sufficient training and experience in using the device
2	Ability of the device to produce reliable measurements
3	Convenience of use and comfort of the device

### Sufficient training and experience in using the device

The Pinard fetoscope has been available and commonly used for decades. Midwives have gained experience in how to use it and awareness of its strengths and limitations. It also appears that knowledge and skills of using a particular device build over time. Hence, those who were exposed during midwifery training in using a device, and continued to use the same device in their work, tend to prefer that device.

*R2. …I have gained enormous experience using Pinard. I have used it for many years, since when I was studying in nursing college…. [FGD1].*

*R4. …We have been using Pinard for many years, and we have helped many women with it…. [FGD4].*

*R5…. I can easily monitor fetal heart rate and discover abnormalities by using Pinard because I am used to it. I have used it for a very long time, and I know it well. I can easily differentiate abnormal from normal fetal heart rates when I auscultate using Pinard…. [FGD3].*

Midwives admitted that they had less experience using the Freeplay Doppler. They were neither taught how nor required to use a Doppler during midwifery training. Furthermore, midwives claimed that they had insufficient on-job training when the Doppler was introduced in the hospital, as the quotes below illustrate:

*R1. … We were not trained to use Doppler during midwifery training, and the on-job training was not enough for us to be thoroughly competent in using Doppler. …. Doppler is good only if people are sufficiently trained. Adequate Doppler training is necessary for every midwife because we were trained only with Pinard during midwifery training. …Doppler came in the labor ward recently, and most of us are not used to it. [FGD3].*

*R3…. Sufficient Doppler use training is necessary to make us knowledgeable and up to date…. [FGD5].*

### Ability of a device to produce reliable measurements

The device’s ability to produce reliable measurements was delineated to influence midwives’ preferences. Participants much preferred the device they believed to produce the most reliable measurements over the one they believed to possibly produce incorrect measurements. Midwives perceived the two devices’ ability to produce reliable measurements differently, as demonstrated in the following quotes:

*R5. …. When I auscultate fetal heart rate using Pinard, I hear the fetal heart beats with my own ears, whether they are slow, fast, or within a normal range. Most of the time when using Pinard, I am certain about it (FHR), because I hear it myself. ….. [FGD1].*

*R4. …. I trust Pinard, because I hear the fetal heart rate myself. I can easily know whether it is normal or abnormal…… [FGD3].*

Personal hearing and counting the FHR were perceived by midwives as important aspects of feeling confident about the measurements when using the Pinard fetoscope. In addition, some midwives found it easy to discover FHR abnormalities.

*R2. …When I use Pinard, I hear and count fetal heart rates myself; I can be sure they are fine. There is no mistake, because I have counted them myself……. [FGD4].*

On the other hand, Freeplay Doppler results were perceived as reliable by some midwives, due to the Doppler’s ability to display the FHR number and produce sound. This seemed to make some midwives confident of the measurements produced by the Freeplay Doppler.

Both hearing the rhythm and seeing the FHR number on the Doppler display were mentioned as a benefit, contrary to the Pinard, which does not display the FHR number or produce a sound.

*R1...When I appropriately place Doppler on a mother’s fundus, I can auscultate the fetal heart rate for the whole minute... [FGD5].*

*R4…. Doppler displays the number and gives sound of the fetal heart; even if I cannot read the number, I can hear the sound. This makes me trust Doppler…. [FGD1].*

*R5…. The mother can also hear the heart sounds of her fetus when auscultated using Doppler, and if she is able to read can read from the display……. [FGD3}.*

Some midwives raised concerns about the way FHR was assessed using the Pinard, because personal auscultation, counting using a watch, and calculations may lead to incorrect measurements:

*R1. Two of us may be auscultating the fetal heart rate of the same fetus using Pinard, one after another. I can say they are normal and my fellow says they are abnormal…. [FGD1].*

*R5… Sometime when I am in hurry, I fail to count them correctly and identify if they are abnormal. It is difficult to discover it quickly with Pinard. But with Doppler, it displays the measurements quickly. It is easy to see if the heart rates are low or high, and I can repeat auscultation. But with Pinard, I can’t easily repeat…. Pinard is difficult to use in a busy ward.… FGD4].*

*R3. …. I think Pinard is good, but the measurement produced by Pinard depends on the individual’s ear, as she is the one who hears and counts the beats…. [FGD3].*

On the other hand, Doppler was perceived as prone to incorrect measurements, especially when the capacity of the battery was low, which was typically after being used many times without proper charging.

*R4…. Doppler can produce wrong measurements when it has low charge; I doubt it sometimes, because it needs to be well charged to produce true measurements…. [FGD5].*

*R2. …Doppler can give wrong measurements if is used to monitor many women without proper charging in between. ……. [FGD4].*

### Convenience of use and comfort of a device

The midwives preferred the device which they perceived as easy to use, always usable, and possible to control, rather than complex devices. Midwives perceived the Pinard fetoscope easy to use even in areas without electricity, and it is possible to “personalize” the findings by “hiding the findings” from others including the mother. The Pinard fetoscope neither displays the number nor produces any sound. For some midwives, this was considered important to avoid panic among mothers, especially when the fetus was showing signs of demise. The quotes below are illustrative:

*R3…. When I use Pinard, I find it simple as I just pick it up and start using it. I don’t have to charge it or look for a battery; it is always ready for use… [FGD2].*

*R5…. Pinard is ready for use all the time, not like Doppler, which needs electricity to charge. For Pinard, there is no need of electricity; it is ready for use all the time…. [FGD2].*

*R3. …. Pinard has confidentiality to some extent, not like Doppler, which produces sound and displays measurements. Amid many women, others can hear or read the fetal heart rate of another woman and say it is not normal; this may lead to panicking, ……. but with Pinard, once I know beats are not ok, I will know how to counsel the mother, without any disturbances from other laboring women……. [FGD5].*

The Freeplay Doppler’s ability to display FHR numbers, and to be used during contractions and in the second stage of labor, were perceived by the midwives as convenient attributes. Midwives also cited the ability to conveniently monitor many women without fatigue from bending over:

*R1. … Doppler displays the fetal heart rate and gives sound immediately when appropriately placed on the fundus; there is no delay and no need to count. I just read the numbers; for me it (Doppler) is good…. [FGD4].*

*R5…. Doppler displays the fetal heart rate; I can see and know for sure when it is high, low, or within the normal range. It is good because I see with my eyes…… [FGD3].*

With the Pinard fetoscope, the midwife must bend over the mother, press a bit hard on the mother’s abdomen, and have access to a watch to be able to count the FHR. These factors were considered slightly negative attributes for the convenience of the Pinard fetoscope. Difficulties in sharing the findings, causing additional pain to the women, and the challenges in use during the second stage of labor, were also raised by midwives as negative attributes for the convenience of using the Pinard fetoscope:

*R4. It is very difficult to use Pinard during the second stage since the mother has strong contractions…. sometimes we estimate the FHR or measure after contractions, but for women not in the second stage of labor Pinard is good… [FGD2].*

*R5. Pinard is easy to use when contractions are over; that’s when I can easily get the fetal heart rate… but when the mother has contractions, I cannot measure fetal heart rate properly, because the abdomen is tense…. [FGD2].*

*R1. Some women throw it (the Pinard fetoscope) away even before 1 min, and I cannot get the correct fetal heart rate per minute, … When I use Pinard, she throws it away …… she says, “it is painful; take it away.” [FGD3].*

The perceived downsides of Freeplay Doppler with respect to convenience of use were the need for electricity or cranking to be fully charged, the time needed for charging, and the need for jelly to properly measure the FHR:

*R3. It is difficult to use Doppler when there is no electricity; it is easy to use Pinard, which does not need electricity. ….. [FGD3].*

*R1 …. Doppler is a machine, which needs electricity; at some places it can’t be used because there is no electricity, and without it being charged you can’t use it…… that is a limitation of Doppler. [FGD3].*

*R2. …. Doppler needs jelly; it is difficult to use it if there is no jelly. [FGD1].*

## Discussion

The midwives participating in these FGDs had different views regarding the two devices (Pinard fetoscope and Freeplay Doppler), and the FGDs did not reveal a common clear predilection for one of the devices. Based on their opinions, three main themes emerged as important factors affecting user preference; 1) sufficient training and experience in using a device, 2) the perceived ability to produce reliable (accurate) measurements, and 3) the convenience of use and comfort of the device.

Fetoscopes are the most common devices used to monitor FHR in low-resource settings, much more common than Dopplers. Fetoscopes have been in use for decades, need no electricity, and are highly portable and relatively inexpensive; hence, they are easily available for use in both training institutes and facilities in low-resource settings [27]. Midwives clearly pointed out that having adequate pre-service training and many years of clinical experience in using fetoscopes made them feel confident; thus, they preferred to use this device. Knowledge is acquired over time, and some midwives said they trusted the Pinard fetoscope more than the Freeplay Doppler because they had more experience in using the Pinard fetoscope. They were more confident about their own auscultations and measurements using the Pinard fetoscope than they were simply watching the numbers produced by the Freeplay Doppler. However, they emphasized that this uncertainty was likely caused by inadequate training, knowledge, and experience in using the Freeplay Doppler.

These findings are discussed in relation to innovation/diffusion theory, per Rogers (2003) [28], which seeks to explain how, why, and at what rate new ideas and technology are spread and adopted [29]. Rogers categorizes adopters of a new device/technology into five categories: Innovators, Early adopters, Early majority, Late majority, and Laggards. Innovators are those who usually lavish much time, energy, and creativity on developing the new idea/device. Early adopters leap in once benefits are obvious, and they are quick to make connections with their personal needs. Early majority are pragmatists, comfortable with moderately progressive ideas, but needing solid proof of benefits. Late majority are not risk takers and are uncomfortable with new ideas; change is difficult for them. They need adequate training and longtime use of an idea/device to take it in. Laggards hold out to the bitter end with old devices because they see high risk in adopting a new device/technology [30]. Based on the theory of diffusion (innovation theory), the midwives’ opinions show that they fit into Early adopters, Early majority, Late majority, and Laggards categories of how people usually accept a new product (Freeplay Doppler in our case). Those with long experience in the labor ward fit into the category of “Laggards,” i.e., they were traditional and conservative, preferring the Pinard fetoscope over the Freeplay Doppler simply because they had used it for a long time. They admitted that the Pinard fetoscopes’ measurements were subjective, and that it was difficult to use during the second stage of labor. Some midwives qualified for the “Late majority” category because they were skeptical of change and had persistent doubts that the Doppler was prone to errors [31]. Sufficient training and a substantial period of Freeplay Doppler use is necessary to overcome their skepticism.

Mahomed et al.(1994) and Byaruhanga et al. (2015) found that a hand-held Doppler detected FHR abnormalities more reliably than the Pinard fetoscope [11, 13]. In our study, some midwives’ concerns were the accuracy/reliability of Freeplay Doppler measurements when it was frequently used or when not fully charged. Most midwives valued the ability to easily communicate and share the FHR when using the Freeplay Doppler compared to the Pinard fetoscope. Many midwives perceived the Pinard fetoscope as producing inaccurate measurements most of the time, despite its long-term use. With the Pinard fetoscope, the midwife auscultates using her ears and counts beats against a clock, which makes the measurements subjective and prone to error. Some midwives admitted that they usually counted for shorter time periods and multiplied, which may lead to even more incorrect measurements. The midwives’ concerns about the Pinard fetoscope concur with Lewis et al.(2015) the International Federation of Gynecology and Obstetrics consensus guidelines on intrapartum fetal monitoring [27]. The report indicates that midwives want a device they can trust all the time. Smith et al. also found that professionals wanted to be certain about the FHR findings and to be able to communicate their findings adequately [20]. Unfortunately, both devices have shortcomings; they are not very accurate as perceived by midwives.

A device is perceived to be convenient for use if it is easy to use and understand. The Pinard fetoscope, being an inexpensive wooden device, which is always available for use without power and does not require much knowledge to operate, was considered by many midwives to be convenient for use. Its disadvantage is the need of a watch to be able to count correctly. On the other hand, the Freeplay Doppler was preferred by some midwives because of its ability to display a number and produce a sound. Ready availability, display of numbers, and sound production also make a device convenient to use, per midwives’ opinions. In addition, a device which was perceived to be comfortable for both midwives and mothers motivated the midwives to adequately monitor FHR during labor. In this study, midwives clearly aired that using the Pinard fetoscope could be painful for the mother, especially during auscultations when some compression on the abdomen is necessary to be able to hear the FHR. Furthermore, the midwives must bend towards the mothers to be able to listen. When there are many women to monitor, using the Pinard fetoscope was considered by midwives to be a wearisome task and demotivating situation. These findings concur with a study done by Mangesi et al., that assessed the mothers’ preferences on FHR monitoring devices, which found that intermittent auscultation of the fetal heart during labor with a Doppler was more acceptable to laboring women than monitoring with a Pinard fetoscope or a cardiotocograph [15].

### Strengths of this study

The study managed to get detailed information about feelings and perceptions of both groups and individual midwives through the FGDs. Our study was part of the larger Safer Births project, and the findings explain the gaps in the previous randomized study, which found many crossovers, with the Pinard fetoscope being used more than the Freeplay Doppler [19]. FGDs were moderated by three moderators with different backgrounds (an obstetrician, a senior nurse midwife, and a clinical psychologist). This mix of backgrounds and professional experiences made it easy to capture most of the study objectives, and the midwives were free to express their opinions. Both manual records and a computer software (NVivo 11 program) were used during analysis, making the analysis exhaustive enough to capture all aspects of the discussion.

### Limitation of the study

A single site was represented in our study, with a group of midwives working under similar conditions, so the information may not be able to be generalized to other settings. There was a high turnover of midwives and challenging to retain skilled midwives. This made it necessary to conduct frequent on-the-job trainings, however, it may have influenced midwives’ opinion, perceptions and attitude towards the two devices.

To ensure trustworthiness, this study adhered to Guba’s four criteria of trustworthiness of qualitative studies [31, 32], which are credibility, dependability, transferability, and confirmability. *Credibility* depends on the richness of the information gathered rather than quantity. To ensure credibility, midwives with different working experiences who were working in the labor ward for at least 6 months prior to the study were recruited. Recruitment was done by the first author (PM) and the labor ward’s senior nurse, for the purpose of recruiting suitable midwives who had the potential to give rich information. The FGDs were conducted by PM (who is an obstetrician at the hospital), a clinical psychologist at the hospital, and the last author (CK) (who is a senior nurse midwife from outside the hospital). All these three are native Tanzanians fluent in the Kiswahili language, which was the language used during the FGDs and the mother tongue of the participants. This mix of moderators and use of Kiswahili ensured the richness of the information from participants and minimized any tendency they might have to give “desired,” rather than honest, answers. *Dependability* points to the fact that the research findings are consistent and could be repeated. To ensure dependability of the study, the FGD guide used open-ended questions. The interview guide directed the flow of the discussion, which ensured that the research questions were explored extensively and answered. *Transferability* describes the degree to which the research can be transferred to other contexts. Our study setting and participants’ characteristics are well described in the methodology section to enable readers to judge if the results could be transferred to their own context with similar characteristics. *Confirmability* questions how the research findings are supported by the data collected. The confirmability of the study results was promoted by a flexible FGD guide, an emergent design [33], a structured analytical procedure, and the presentation of quotes from the informants. Five of the co-authors are native Swahili speakers, which made it easy to review the transcripts, both in Kiswahili (the original language of the FGD) and English; this ensured consistence of the data.

## Conclusion

The midwives had mixed opinions on the use of the Pinard fetoscope versus the Freeplay Doppler for FHR monitoring during labor. The main factors affecting the midwives’ perception and preferences to use a device were sufficient training and experience in using the device, ability of the device to produce reliable measurements, and convenience of use and comfort of the device. There should be regular trainings to make the use of Doppler easier, moreover both fetoscopes and Dopplers should be equally available in all labor wards to make students and midwives gain experience in using both devices for the benefit of the mother and her fetus. It is important to consider midwives’ perceptions when a new device is introduced in labor management to ensure acceptability of the device. More research need to be conducted in other settings to help address the underuse of new devices for FHR monitoring other than fetoscopes.

## Additional file

Additional file 1: The Focused Group Discussion guide. (DOCX 12 kb)

## Abbreviations

CM: Columba Mbekenga
CTG: Cardiotocograph
FGD: Focused group discussion
FHR: Fetal heart rate
HLH: Haydom Lutheran Hospital
MD: Medical doctor
NIMR: National institute of medical research
PhD: Doctor of philosophy
PM: Paschal Mdoe
R: Respondent
REK-vest: Regional committee for medical and health research ethics western Norway
