# Supplementary material for: Midwives’ perceptions on using a fetoscope and Doppler for fetal heart rate assessments during labor: a qualitative study in rural Tanzania
Source: BMC Pregnancy Childbirth. 2018 Apr 16;18:103. doi: 10.1186/s12884-018-1736-y (PMC5902983; doi:10.1186/s12884-018-1736-y)
Supplement: Supplementary file 1 — The Focused Group Discussion guide. (DOCX 12 kb) [file 12884_2018_1736_MOESM1_ESM.docx]

**FOCUSED GROUP DISCUSION GUIDE FOR THE STUDY TITLED “Midwives’ perceptions on using a fetoscope and Doppler for fetal heart rate assessments during labor: A qualitative study in rural Tanzania”**

Welcome and thank you for being here today. The purpose of this gathering is to get your opinions and views regarding intrapartum fetal heart rate monitoring using Pinard fetoscope and hand held Doppler. Specifically, we want to understand your perceptions as midwives on fetoscope and Doppler use for FHR assessments. This will help to address and bridge the gap of human factor associated with the choice of using one device in the presence of other device, here we talk of Fetoscope and Doppler. We understand that you have been using these two devices for more than two years now, and that everyone has her/his preference and reasons to prefer that device. As you all are aware that FHR monitoring is an important component of labor management, we need to know the gaps and challenges to be able to improve this component.

In this discussion we will not use names, as we have given you numbers, we will refer to each one using numbers to hide your identity as we are recording our discussion.

I will be moderating this FGD with my colleague, please each one of you feel free to share your opinions, and each opinion/view is respected and equally valued. Speak freely and loudly.

You are all welcome.

To begin our discussion, please tell us your overview about FHR monitoring during labor?

- Tell us your experience of FHR monitoring during labor

- What components of FHR you normally look for when auscultating FHR

- Quantity

- Quality

- How frequent do you monitor?

- Why?

- What if you don't do that frequent?

- Do you always manage to auscultate that frequent?

What devices have you ever used to monitor FHR?

**About Pinard**

- Pinard Fetoscope has been used to monitor FHR during labor for long time, what is your opinion on its effectiveness. Probe for more

- What are the goodness of this device? Probe for more

- What are the difficulties of this device? Probe for more

**About Doppler**

- For more than two years, you have been using Doppler for FHR monitoring during labor, what is your opinion regarding its effectiveness. Probe for more

- What are the goodness of this device? Probe for more

- What difficulties you face when using Doppler to monitor FHR? Probe for more

DEVICE MOST PREFERED

What devices between Pinard and Doppler do you prefer more to use in auscultating FHR during labor? Why?

Do you trust this device? Why?

*Thank you all for your participation.*
